# Howler monkeys are the reservoir of malarial parasites causing zoonotic infections in the Atlantic forest of Rio de Janeiro

**DOI:** 10.1371/journal.pntd.0007906

**Published:** 2019-12-09

**Authors:** Filipe Vieira Santos de Abreu, Edmilson dos Santos, Aline Rosa Lavigne Mello, Larissa Rodrigues Gomes, Denise Anete Madureira de Alvarenga, Marcelo Quintela Gomes, Waldemir Paixão Vargas, Cesare Bianco-Júnior, Anielle de Pina-Costa, Danilo Simonini Teixeira, Alessandro Pecego Martins Romano, Pedro Paulo de Abreu Manso, Marcelo Pelajo-Machado, Patrícia Brasil, Cláudio Tadeu Daniel-Ribeiro, Cristiana Ferreira Alves de Brito, Maria de Fátima Ferreira-da-Cruz, Ricardo Lourenço-de-Oliveira

**Affiliations:** 1 Laboratório de Mosquitos Transmissores de Hematozoários, Instituto Oswaldo Cruz, Fiocruz, Rio de Janeiro, RJ, Brazil; 2 Laboratório de comportamento de insetos, Instituto Federal do Norte de Minas Gerais, Salinas, MG, Brazil; 3 Divisão de Vigilância Ambiental em Saúde, Secretaria de Saúde do Rio Grande do Sul, Porto Alegre, RS, Brazil; 4 Laboratório de Pesquisa em Malária, Instituto Oswaldo Cruz, Fiocruz, Rio de Janeiro, RJ, Brazil; 5 Centro de Pesquisa, Diagnóstico e Treinamento em Malária, Instituto Oswaldo Cruz, Fiocruz, Rio de Janeiro, RJ, Brazil; 6 Laboratório de Malária, Instituto René Rachou, Fiocruz, Belo Horizonte, MG, Brazil; 7 Grupo de Pesquisa e Epidemiologia Espacial, Departamento de Endemias Samuel Pessoa, Escola Nacional de Saúde Pública Sergio Arouca, Fiocruz, Rio de Janeiro, RJ, Brasil; 8 Laboratório de Doenças Febris Agudas, Instituto Nacional de Infectologia Evandro Chagas, Fiocruz, Rio de Janeiro, RJ, Brazil; 9 Faculdade de Medicina de Teresópolis, Centro Universitário Serra dos Órgãos, UNIFESO, Teresópolis, RJ, Brazil; 10 Núcleo de Atendimento e Pesquisa de Animais Silvestres, Universidade Estadual de Santa Cruz, UESC, Ilhéus, BA, Brazil; 11 Secretaria de Vigilância em Saúde, Ministério da Saúde, Brasília, DF, Brazil; 12 Laboratório de Patologia, Instituto Oswaldo Cruz, Fiocruz, Rio de Janeiro, RJ, Brazil; Vienna, AUSTRIA

## Abstract

**Background:**

Although malaria cases have substantially decreased in Southeast Brazil, a significant increase in the number of *Plasmodium vivax*-like autochthonous human cases has been reported in remote areas of the Atlantic Forest in the past few decades in Rio de Janeiro (RJ) state, including an outbreak during 2015–2016. The singular clinical and epidemiological aspects in several human cases, and collectively with molecular and genetic data, revealed that they were due to the non-human primate (NHP) parasite *Plasmodium simium;* however, the understanding of the autochthonous malarial epidemiology in Southeast Brazil can only be acquired by assessing the circulation of NHP *Plasmodium* in the foci and determining its hosts.

**Methodology:**

A large sampling effort was carried out in the Atlantic forest of RJ and its bordering states (Minas Gerais, São Paulo, Espírito Santo) for collecting and examining free-living NHPs. Blood and/or viscera were analyzed for *Plasmodium* infections via molecular and microscopic techniques.

**Principal findings:**

In total, 146 NHPs of six species, from 30 counties in four states, were tested, of which majority were collected from RJ. Howler monkeys (*Alouatta clamitans)* were the only species found infected. In RJ, 26% of these monkeys tested positive, of which 17% were found to be infected with *P*. *simium*. Importantly, specific single nucleotide polymorphisms–the only available genetic markers that differentiate *P*. *simium* from *P*. *vivax*–were detected in all *P*. *simium* infected *A*. *clamitans* despite their geographical origin of malarial foci. Interestingly, 71% of *P*. *simium* infected NHPs were from the coastal slope of a mountain chain (Serra do Mar), where majority of the human cases were found. *Plasmodium brasilianum/malariae* was initially detected in 14% and 25% free-living howler monkeys in RJ and in the Espírito Santo (ES) state, respectively. Moreover, the malarial pigment was detected in the spleen fragments of 50% of a subsample comprising dead howler monkeys in both RJ and ES. All NHPs were negative for *Plasmodium falciparum*.

**Conclusions/Significance:**

Our data indicate that howler monkeys act as the main reservoir for the Atlantic forest human malarial parasites in RJ and other sites in Southeast Brazil and reinforce its zoonotic characteristics.

## Author summary

The present work comprises an unprecedented capture effort and large-scale field survey of *Plasmodium* species in non-human primates (NHPs) in RJ, a state recording three-decade history of autochthonous human cases of benign tertian malaria lacking epidemiological clarification of their origin. This is the first study to describe the infection rates by *Plasmodium* spp. in free-living NHPs in RJ, thereby matching the spatial distribution of *P*. *simium* in NHP with that of the local human cases of benign tertian malaria occurring due to this parasite. This study confirmed howler monkeys as the only reservoir of this zoonotic malarial parasite in RJ and reported that specific single nucleotide polymorphisms were present in all *P*. *simium* infected howler monkeys, despite their geographical origin of malarial foci. Moreover, this is the first study to record *P*. *brasilianum/malariae* in free-living NHPs from RJ, and to illustrate their widespread distribution in this state. Collectively, these findings help us in evaluating the simian malaria prevalence in the Atlantic Forests and in assessing the zoonotic characteristics of autochthonous human malaria in Rio de Janeiro, thus providing assistance in shaping surveillance and control.

## Introduction

In Brazil, more than 99% of malarial infections are acquired from the Amazon, and few isolated cases are occasionally found and recorded in regions outside the Amazon [1]. Malaria transmission was considered eradicated from South and Southeast regions of Brazil approximately 40 years ago [1]; however, in the last three decades, a significant increase in autochthonous malarial cases by *Plasmodium vivax*-like parasites in the Atlantic Forest areas in Southeast Brazil, where no index case that could have introduced the parasite from a malaria endemic region, has been reported [1,2]. These cases present similar parasitological, clinical, and epidemiological characteristics, such as low parasitemia, no *P*. *vivax* expected relapses, and recent visits to dense rain forest areas, where the bromeliad-inhabiting *Anopheles* mosquitoes belonging to the subgenus *Kertezsia*, specially *An*. *cruzii*, are found [2–4]. *An*. *cruzii* is the main vector of “bromeliad malaria”, which is endemic in South and Southeast Brazil, and is the only known natural vector of simian malaria in the country [5]. This particular epidemiological context revived the hypothesis raised by Deane et al. in the 1960s regarding the existence of human malaria cases of simian origin in Brazil. Specifically, these authors reported a human natural infection attributed to the Neotropical primate parasite *P*. *simium* Fonseca [6] in São Paulo (SP), Southeast region [7]. The patient presented a benign tertian malaria after being exposed to the mosquito bites during a tree-canopy entomological survey in a forest densely populated by *An*. *cruzii*. The description of vertical movement of *An*. *cruzii* between the canopy and ground level in the Atlantic rain forest of Southern Brazil reinforced Deane’s hypothesis that part of the transmission of bromeliad malaria in Southern and Southeastern Brazil would be of zoonotic character, with monkeys being the parasite reservoir [5–8].

Two species of *Plasmodium* have been described in the Neotropical non-human primates (NHP): *P*. *brasilianum* Gonder e Berenberg-Gossler (1908) and *P*. *simium*, almost indistinguishable from the human malaria parasites *P*. *malariae* and *P*. *vivax*, respectively [5,6,9]. Besides subtle morphological variations [2,5], molecular markers such as microsatellites and single nucleotide polymorphisms (SNPs) were the only differences so far described between *P*. *malariae* and *P*. *brasilianum*, and *P*. *vivax* and *P*. *simium* [2,10,11]. *P*. *brasilianum* is widely distributed compared to *P*. *simium*, with its presence from México to Southern Brazil, thereby infecting at least 11 genera of the five families of Neotropical primates (Aotidae, Atelidae, Callitrichidae, Cebidae, and Phiteciidae) [5,12–15]. In contrast, *P*. *simium* has been found essentially in species belonging to two genera (*Alouatta sp*. and *Brachyteles sp*., family Atelidae) [5], from the Atlantic forest of South and Southeast Brazil.

To completely understand the epidemiology of recent autochthonous malaria in Southeast Brazil, it is necessary to confirm the circulation of NHP *Plasmodium sp*. in the transmission foci as well as to determine the parasite reservoirs. Studies on the prevalence of *P*. *simium* and *P*. *brasilianum* infections in NHPs and their potential reservoirs in Southeast Brazilian states were conducted during 1960–1990s. Almost 800 NHPs were sampled and their blood slides were examined by microscopy, which is a less sensitive technique compared to the molecular assays, thus recording a variation in the *Plasmodium* infection from 10.9% in the states of Espírito Santo to 56.5% in SP [5]. During these surveys, only free-living lion-tamarins (Callitrichidae) were examined within RJ and all were negative to malaria parasites [16]. However, RJ recorded 110 autochthonous human cases of benign tertian malaria between 2005 and 2018, with an outbreak in 2015–2016 of 49 cases [1,2]. Interestingly, all these human infections were acquired at the RJ sites located along Serra do Mar, an extensive mountain chain covered by the best-preserved rain forest mosaic in Southeast Brazil. This biome harbors a rich NHP fauna comprising species of six genera (*Alouatta*, *Brachyteles*, *Callicebus*, *Callithrix*, *Leontophitecus*, and *Sapajus*) [17], with *An*. *cruzii* being the most common anopheline mosquito [18]. Consequently, the hypothesis of simian origin in these RJ malarial cases has been raised [5,7]. In response, multidisciplinary malaria studies including clinical, epidemiological, parasitological, and molecular approaches have been conducted in RJ [2,4,19]. More recently, the molecular studies of parasites infecting humans and three howler monkeys clearly demonstrated that they shared the same *P*. *simium* parasite [2]; however, to date, scarce number of wild NHPs of few species from only three out of numerous autochthonous malaria foci in RJ and surroundings could be examined [2]. This study presents the largest sampling effort ever carried out in the Atlantic forest of RJ and its borders for capturing and examining free-living NHPs in order to describe the geographical distribution and frequency of simian malaria, as well as to determine the local animal reservoirs and confirm the identity of the parasite infecting humans and NHPs in the autochthonous malaria foci.

## Material and methods

### Study area

The work was carried out between May 2015 and January 2019, with a total of 120 days of fieldwork at 44 sites in 30 counties of the Atlantic Forest biome, mainly in RJ but also in its bordering areas including the states of Minas Gerais (MG), ES, and SP. In this survey, we included forest fragments from lowlands areas to mountain valleys and escarpments of mountain chains such as Serra do Mar, which divides the state territory into two sides, one facing the ocean (hereinafter called the coastal slope) and other the continent (continental slope) [20,21]. The choice of capture areas considered the local existence of NHPs, recent human malaria cases as well as alerts from the information network built with key institutions to continuously monitor the presence of howler monkeys, as previously described [21].

### Capture and sample collection

The expeditions included up to 10-day surveys in the forests, conducted by 2–6 trained people in the target areas to search NHPs. The capture method was selected according to the NHP species, behavior, and size [22]. Briefly, Tomahawk model traps baited with banana were used for *Callithrix*, *Leontopithecus*, and *Sapajus* genera [22,23]. The traps were opened early during daytime and were inspected every hour until 3:30 pm, when they were closed. The captured animals were anesthetized with ketamine (15 mg/kg) + xilazine (0.5 mg/kg). Anesthetic darts containing ketamine (15 mg/kg), midazolam (1 mg/kg), and levomepromazine (1 mg/kg), or alternatively, a combination of tiletamine and zolazepam (4–5 mg/kg) were used for the *Alouatta* and *Brachyteles* genera, as well as for one titi monkey (*Callicebus*) [21,24]. Sick animals reported by the information network during the 2017–2018 yellow fever epizooties [25] were captured with nets [21]. A sample of 3–6 mL of blood was collected from the anesthetized or recently dead animals. Thick and thin blood films were immediately prepared, and the remaining blood was allowed to coagulate. After collections and the complete recovery from anesthesia (2–3 h), they were released to their habitats during the daytime from where they were captured. Liver samples were obtained from dead animals, which recently died due to yellow fever or any other disease. Liver and blood samples were stored at −80°C until DNA extraction. Importantly, only one monkey was injured during the fall post anesthesia, which was then treated and kept in a primate-breeding center (Centro de Primatologia do Rio de Janeiro—CPRJ) [21].

### Malaria diagnosis

Giemsa’s solution stained thick and thin blood films were examined under a microscope with a 100× oil-immersion objective by two trained and independent microscopists. DNA was extracted from the blood clots as previously described [26] and from the liver samples [27] using the QIAGEN DNeasy mini kit according to manufacturer’s instructions. Molecular diagnosis was made via conventional PCR. All DNA samples were tested in triplicate for 18S rRNA *Plasmodium* genus-specific gene [28,29], and then for cysteine proteinase *P*. *vivax* and ssrRNA *P*. *malariae* and *P*. *falciparum* genes, as previously described [29,30]. The sensitivity thresholds of the protocols used were 0.5, 0.019, 1.0, and 1.0 parasite per μL for the *Plasmodium* genus, *P vivax*, *P*. *malariae*, and *P*. *falciparum* assays, respectively [28–30]. Moreover, an internal control (betaglobin primers) was used to assess eventual enzyme inhibitors that could generate false negative results and all the samples generated betaglobin amplicons.

*P*. *vivax-*positive samples were subjected to *P*. *simium* differential diagnosis based on a mitochondrial SNP, the only genetic marker available to differentiate them [2,10]. The molecular diagnosis was performed via nested-PCR of *coxI* gene fragment and subsequent enzymatic digestion, using primers and a previously described protocol, which detected 3.12 parasites/μL [10]. All the PCR products were visualized under UV light after electrophoresis on 2% agarose gels.

### Histopathological analysis

Spleen fragments of a subsample comprising 16 howler monkeys (12 from RJ and 4 from ES), presumably found dead due to yellow fever, were fixed in Carson’s formalin-Millonig [31] and further processed according to the standard histological techniques for paraffin embedding. Sections (5-μm-thick) from each block were stained with hematoxylin–eosin [32] or Lennert Giemsa [33] and analyzed for malarial pigments under an AxioHome microscope equipped with an HRc Axiocam digital camera (Carl Zeiss, Germany).

### Ethical issues

The collection methods, biosafety, and anesthesia protocols adhered to the Brazilian law (11.794 of July 8, 2008) on the use of animals in scientific research, and complied with the rules and regulations of Brazilian Ministry of Health [22], having been previously approved by the institutional Ethics Committee for Animal Experimentation of Instituto Oswaldo Cruz (protocol CEUA/IOC-004/2015, license L-037/2016) and by Brazilian Ministry of the Environment (SISBIO 41837–3 and 54707–4) and Rio de Janeiro’s Environment agency (INEA 012/2016 and 019/2018). The research also adhered to the American Society of Primatologists Principles for the Ethical Treatment of Nonhuman Primates.

## Results

**Fig 1:**
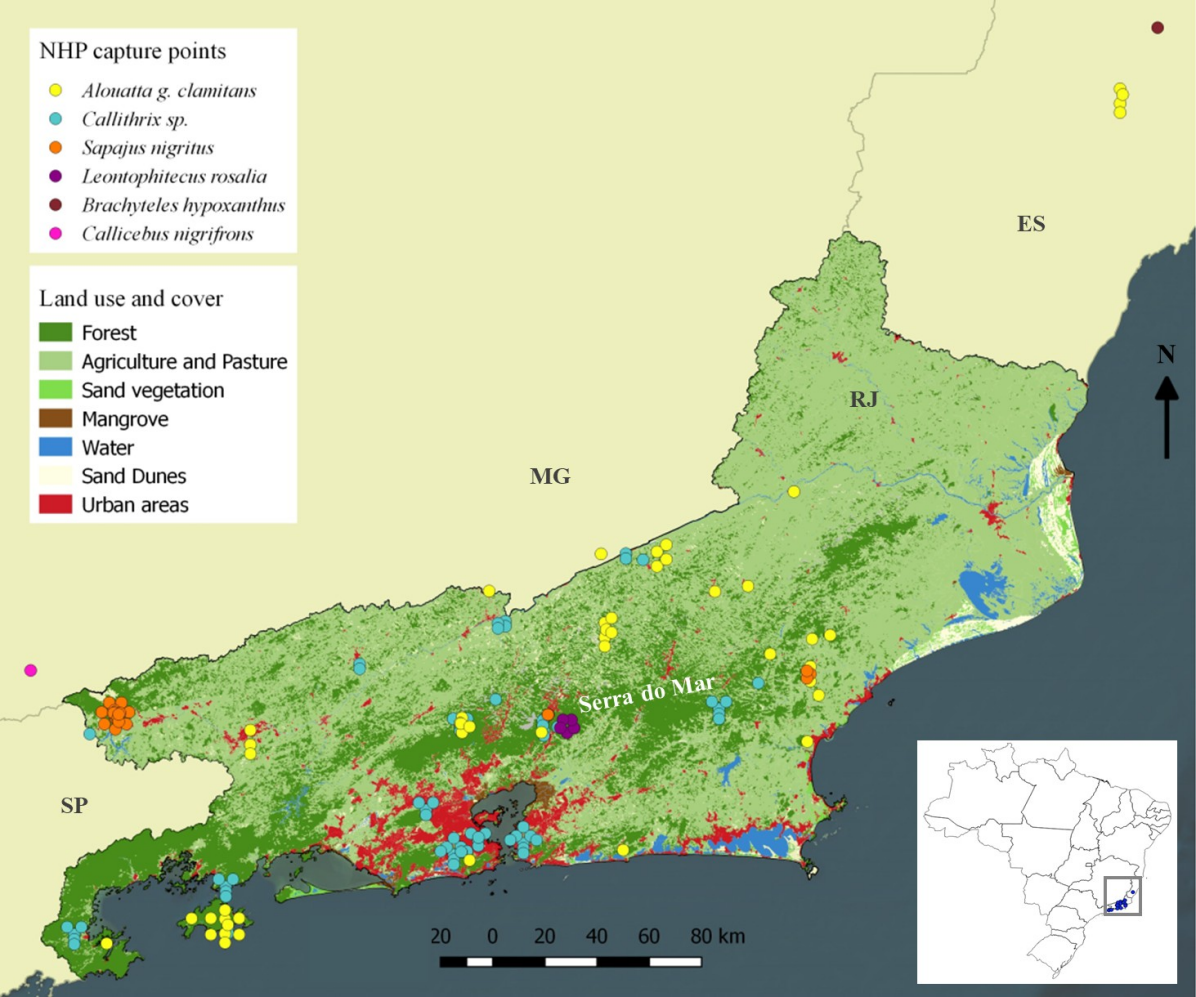
Map presenting collection points of non-human primates in Rio de Janeiro and bordering states in Brazil. Each circle represents one examined NHP. The figure was prepared using free software QGIS 2.18.

In total, we examined 146 animals belonging to six species from 30 counties in four Brazilian states, with majority of animals being from RJ (S1 Table and Fig 1); of these, 130 animals were screened by microscopy and PCR using blood samples, seven by microscopy and PCR from blood samples and histopathology of spleen fragments, and nine by PCR of viscera and histopathology of spleen fragments.

**Fig 2:**
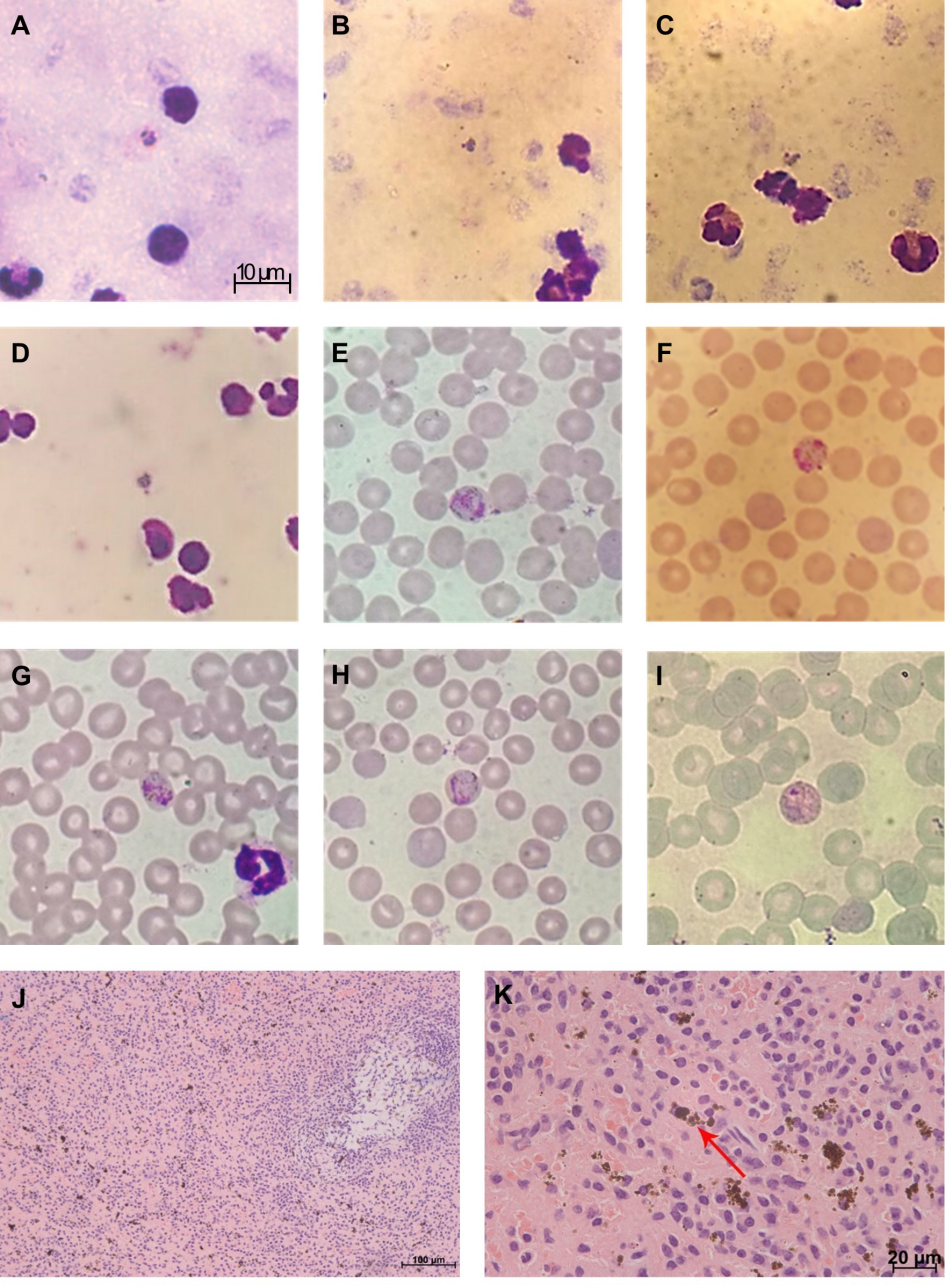
Giemsa’s solution-stained thick (A-D) and thin (E-I) blood samples, and histopathological analysis of hematoxylin-eosin-stained spleen fragments of howler monkeys that were naturally infected with *Plasmodium* in Rio de Janeiro state, Brazil, presenting (J) hypertrophy of red pulp with malarial pigments and white pulp atrophy and (K) details of malarial pigments in the red pulp.

Despite their geographical origin, the only NHP species found to be infected with *Plasmodium* was the howler monkey *Alouatta guariba* *clamitans* (Table 1). The PCR method was more sensitive compared to the microscopic examination of blood films, which eventually failed to detect *Plasmodium* in two infected howler monkeys, one harboring *P*. *simium* and another *P*. *brasilianum/malariae* (Table 1). Nevertheless, the results suggest that infected howler monkeys generally exhibit detectable parasitemia during microscopic examination of blood slides, for both *Plasmodium* species. The parasitemia ranging from 15–300 parasites/μL (median = 40 p/μL). Trophozoites were the most commonly visualized blood forms; however, schizonts and gametocytes were also detected (Fig 2). In addition, by using PCR, we were able to detect both *Plasmodium* species in four animals from liver, spleen and blood samples, which showed concordant diagnostic results.

**Table 1:** *Alouatta g*. *clamitans* captured and examined per state, with the infection rate for each *Plasmodium* species and detection method for the present and previous infections. Number (%).

State	N	Total with *Plasmodium*	*P*. *simium*	*P*. *brasilianum / malariae*	*P*. *simiun* and *P*. *brasilianum*/ *malariae*	*P*. *falcipa-rum*	Diagnosis		Malarial Pigment^$^	
							Blood slides + PCR	Only PCR	N	Positive
**RJ**	42	11 (26.1)	5 (11.9)	4 (9.5)	2 (4.7)	-	7	4*	12	6 (50)
**ES**	4	1 (25.0)	-	1 (25.0)	-	-	NR	1	4	2 (50)
**MG**	2	-	-	-	-	-	-	-	-	-
**TOT.**	**48**	**12 (25.0)**	**5 (10.4)**	**5 (10.4)**	**2 (4.1)**	**-**	**7**	**5**	**16**	**8 (50)**

*Two harboring *P*. *simium* and two with *P*. *malariae/brasilianum*. NR: not realized, as they were found dead during a yellow fever outbreak.

^$^Search in spleen tissues in a subsample comprising dead animals.

Only 12 NHPs were examined from the bordering states of RJ, being two howler monkeys from MG and four from ES and none of these were infected by *P*. *simium*. One of the four examined *A*. *clamitans* from ES was PCR positive for *P*. *brasilianum/malariae* (25%; Table 1).

**Fig 3:**
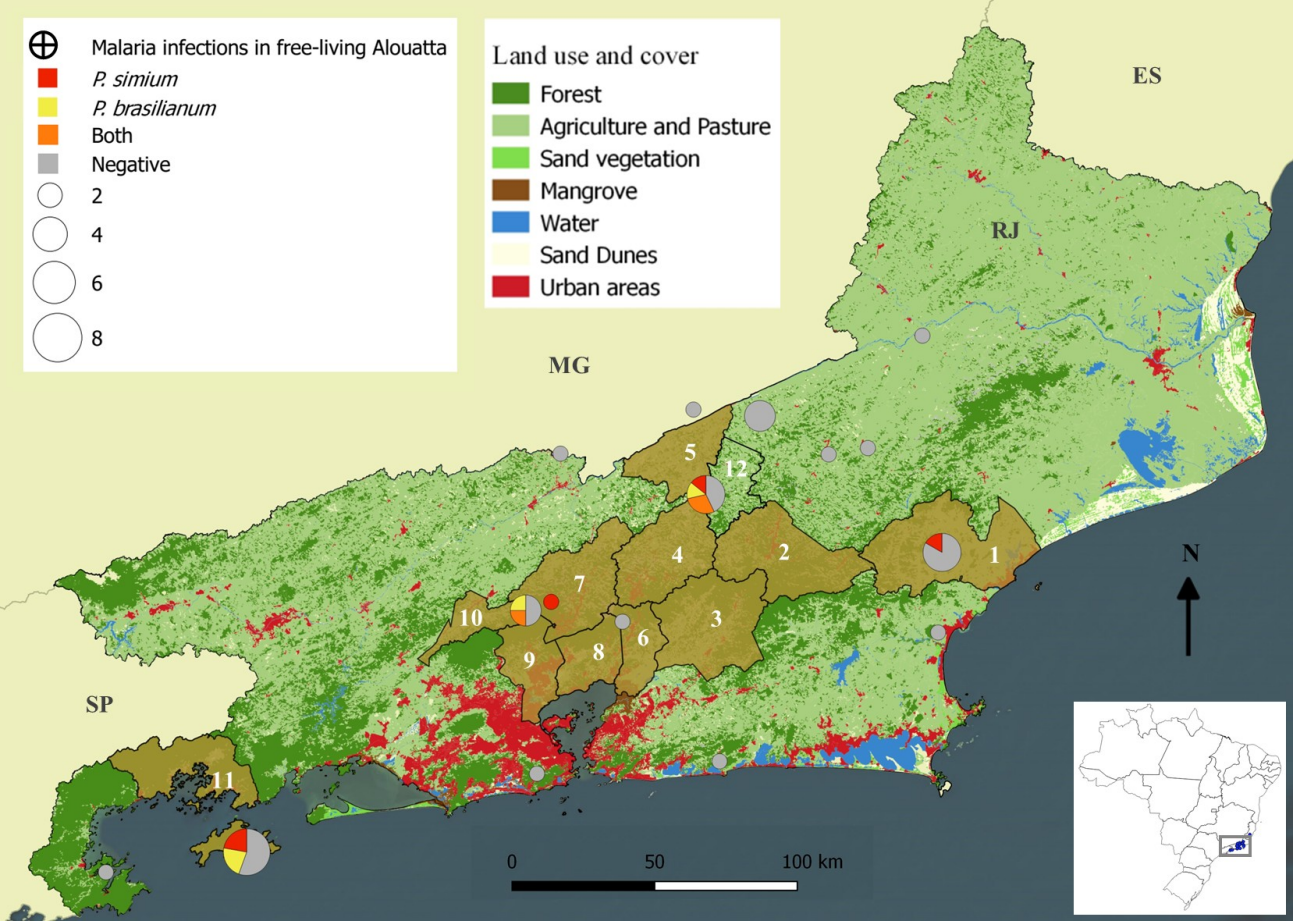
Map presenting the distribution, number, and *Plasmodium* infections of the examined *Alouatta g. clamitans* in Rio de Janeiro. Brown shaded areas represent the counties with registered autochthonous malaria in humans: 1. Macaé, 2. Nova Friburgo, 3. Cachoeira de Macacu, 4. Teresópolis, 5. Sapucaia, 6. Guapimirim, 7. Petrópolis, 8. Magé, 9. Duque de Caxias 10. Miguel Pereira, 11. Angra dos Reis. Number 12 represents Sumidouro, where human cases were not detected. The figure was prepared using free software QGIS 2.18.

Regarding RJ, 11 (26.1%) howler monkeys were infected with malarial parasites during sampling and, among these, seven *(*16.7%) were infected by *P*. *simium*, the causative agent of the autochthonous human malaria in this state (Tables 1 and 2). Importantly, the unique specific *P*. *simium* SNPs used to distinguish *P*. *simium* from *P*. *vivax* were detected in 100% of these tertian malarial parasite infected howler monkeys. Moreover, most of these animals originated from the coastal slope of Serra do Mar, where counties face the ocean and are influenced by its humidity, where several human cases have been recorded in the last decade (Table 2 and Fig 3). Six *A*. *g*. *clamitans* from RJ were infected by the quartan-malarial parasite *P*. *brasilianum/malariae* (14.3%), of which two were co-infected with *P*. *simium* (Tables 1 and 2). All samples were negative for *P*. *falciparum* parasites.

**Table 2:** *Plasmodium*-positive howler monkeys, based on their plasmodial species, county, year, slope of capture, and occurrence of autochthonous human cases of benign tertian malaria, recorded in the respective county and the year of detection in Rio de Janeiro.

*Plasmodium* infections in NHP					Previous NHP *Plasmodium* infection^$^	Human “*vivax*-like” cases	
Serra do Mar Slope	County	NP (%)	Parasitemia (p/mm^3^)	*Plasmodium* species		N	Year
**Coastal**	Miguel Pereira	2 (50)	40 300	*P*. *brasilianum* *P*. *simium + P*. *brasilianum*	0 of 1	10	2015–2017
	Macaé	1 (16.6)	25	*P*. *simium*	3 of 3	12	2011, 2013, 2015–2017
	Petrópolis	1 (100)	0	*P*. *simium*	NA	3	2015–2016
	Angra dos Reis	4 (40)	0 NR 250 NR	*P*. *brasilianum* *P*. *brasilianum* *P*. *simium* *P*. *simium*	1 of 4	3	2015, 2017
**Continental**	Teresópolis*	1 (33.3)	40	*P*. *brasilianum*	NA	0	_
	Sumidouro	2 (100)	15	*P*. *simium*	NA	0	_
			240	*P*. *simium + P*. *brasilianum*	NA	0	_

NP = number of *Plasmodium* positive howler monkeys.

^$^Eight howler monkeys were found dead due to yellow fever virus (YFV), with *Plasmodium*-negative results (PCR and/or blood slides) in three counties where *Plasmodium*-positive howler monkeys were found. Histological preparations of spleen fragments revealed malarial pigment in four (50%) of these PCR-negative animals, suggesting previous infections.

*The *P*. *brasilianum* infection was found in the district of Água Quente, in the continental side of Teresópolis. NA = viscera non available.

Previous malaria infections could be investigated by searching the malarial pigment in a subsample of 16 dead howler monkeys. Accordingly, the malarial pigment (Fig 2) was detected in the spleen fragments of five out of 13 animals with negative PCR at the time of death and, as expected, in three animals with positive PCR (Table 1). Interestingly, this pigment was found in the spleen samples of 50% (eight out of 16) of dead howler monkeys in both RJ (six of 12) and ES (two of four), indicating that malarial parasite is frequent in monkeys from both states.

Howler monkeys were examined from 15 counties in RJ, of which 6 counties presented records of autochthonous human malaria in the last years. Present infections by *P*. *simium* were diagnosed in howlers from four (66.6%) of the surveyed counties reporting human cases of benign tertian malaria in the state, and in a neighboring county (Sumidouro) where the human cases were never detected (Fig 3). Interestingly, in Macaé, where the highest number of human cases was recorded, all dead howler monkeys had malarial pigments in their spleen, suggesting that simian malaria is highly enzootic in that county (Table 2).

## Discussion

The present study demonstrates an unprecedented capture effort and large-scale field survey of plasmodial species in NHPs in RJ, a state recording a three-decade history of autochthonous human cases of benign tertian malaria [1,2]. To our knowledge, this is the first study to describe the NHP infection rates by *Plasmodium*, and match the spatial distribution of *P*. *simium* in NHP with the local human cases previously recorded; howler monkeys were the only confirmed reservoirs of this zoonotic malaria in the state, and the presence of specific SNPs was demonstrated in all *P*. *simium* infected howler monkeys, despite their geographical origin. Although *P*. *brasilianum/malariae* has already been found in captive NHPs from RJ [13], this is the first study to demonstrate that this parasite species was located in free-living NHPs, thereby contributing to its widespread distribution and zoonotic potential in the state.

*P*. *simium* and *P*. *vivax* have similar morphologies [5], immune response [34], and several genetic targets [35,36], for example, PCR based on 18S SSU rRNA, largely used for malaria diagnosis in humans [30], was unable to distinguish these parasites [10,13]. The only genetic markers that can differentiate *P*. *simium* from *P*. *vivax* are the SNPs (3535 T>C and 3869 A>G) in the 6 Kb region of the mitochondrial genome [2]. Excluding the taxonomic issue and focusing on public health impacts, our results confirm that, to date, the *P*. *simium* specific SNP is carried by all parasites isolated from NHPs in the Atlantic Forest, which is in accordance with the study by Alvarenga et al. (2018) [10]. Furthermore, *P*. *simium* was detected in howler monkeys captured in five out of 11 counties recently reporting autochthonous human malaria cases in RJ (Fig 2). Despite numerous efforts, we failed in capturing howler monkeys in four malarial foci due to some local hindrances such as the hunting pressure that scared the monkeys, steep terrains, and low *A*. *g*. *clamitans* population densities [21,37]. Nevertheless, the strong geographical overlap of howler monkey and human infections by parasites displaying specific *P*. *simium* SNPs in five of six malarial foci, strengthens the importance of howler monkeys as the main reservoir of benign tertian human malaria over the zoonotic transmission areas in Southeast Brazil [4,5,38,39]. Howler monkeys have also been by far the NHPs most commonly parasitized with both *P*. *simium* and *P*. *brasilianum* in Southern and Southeastern Brazil [5,40]. Besides their susceptibility to *Plasmodium* infection [5], their acrodendrophilic behavior, huge bodies, and the slower movement through the tree canopies (compared to the smaller monkeys) [41] may make them more prone to mosquito biting vectors. Importantly, the detection of malarial pigment in spleen fragments of eight (50%) of the subsample consisting of 16 howler monkeys from RJ and ES, some of which were *Plasmodium*-negative by PCR at the time of death, suggest that simian malaria is very frequent in this species. Indeed, in some areas with the highest human cases (e.g., Macaé), four out of six (66.6%) howler monkeys were exposed to *Plasmodium* sp., when considering those with present and past malarial infections. This finding may also suggest that spontaneous healing from malarial infections may occur in the howler monkeys found in nature, as observed in the *P*. *simium* experimental infections in certain Neotropical NHPs [42] and in a human natural infection [7].

The frequency of *P*. *simium* infection in free-living howler monkeys in RJ (16.6%) was higher than that previously found in the bordering state of SP (5.8%) [39]; however, it was lower than that found in the entire South and Southeast Brazilian regions (26.3–35%) [5,43]. No previous data are available for comparison in RJ, as this was the first time that howler monkeys were captured in the state in a systematized manner. *A*. *g*. *clamitans* was also the only free-living NHP from RJ in which blood forms by microscopy and plasmodial DNA were detected. Recently, DNA but not blood forms of *P*. *simium* was detected in captive *Cebus* and *Sapajus* from the Southeast [27]. The parasite could be unable to establish the erythrocytic cycle or erythrocytic infection could be transitory, due to unspecificity during cell invasion or host immune competence. Thus, their role as a reservoir for zoonotic malaria in the region remains unclear [27]. *P*. *brasilianum* DNA was detected in captive capuchin, titi, howler, and owl monkeys, as well as in tamarins and marmosets [13,27], most of these being exotic species, that are not found in RJ. All these NHPs were confined in a breeding institution CPRJ located in a well-known simian malarial enzootic transmission area in RJ. Therefore, it was suspected that the local ecological conditions favored the accidental infection of these captive NHPs by parasites carried from infected free-living howler monkeys. Moreover, one free living specimen was infected by *P*. *simium* near CPRJ [27]; however, no evidence is available confirming if the parasite DNA found in the blood of these captive animals implies that they really undergo *Plasmodium* infections or only bear a transient parasitemia. Nevertheless, it is important to continuously monitor their potential role as a zoonotic *Plasmodium* reservoir, besides howler monkeys.

Although *P*. *brasilianum* has been found in several NHP genera [5,44–48] around other Brazilian regions, the previous studies conducted in Brazilian Atlantic forest and Cerrado biomes did not find any capuchin (56 examined) or marmosets (out of 44) infected with *Plasmodium* [39]. Similarly, more than 270 marmosets and lion-tamarins from the Southeast region were *Plasmodium* negative [5]. Splenectomized capuchins remained uninfected when injected with *P*. *simium* infected blood, whereas splenectomized marmoset endured low parasitemia [42]. Thus, the epidemiological role of other NHPs besides howler monkeys in the zoonotic transmission of malaria in Southeast Brazil, including the RJ state, if any, is presumably negligible.

*P*. *brasilianum/malariae* was found in a frequency similar to that of *P*. *simium*, six (14.3%) versus seven (16.7%), respectively–in howler monkeys from RJ, and mixed infections were recorded in two (4.6%) animals. *P*. *brasilianum/malariae* was the only malarial parasite detected in *A*. *clamitans* from ES (Table 1). Besides, the geographical co-occurrence of these parasites seems to be frequent in RJ, as it was revealed in three out of five counties, wherein howler monkeys were detected with the malarial parasites (Fig 2). Interestingly, despite this coincident distribution and similar frequency of *P*. *brasilianum/malariae* and *P*. *simium* in RJ, autochthonous human cases in this state have been diagnosed microscopically and/or molecularly as benign tertian malaria due to *P*. *vivax* for decades [1,5,7,38,49,50]. In particular, *P*. *simium* was only identified by molecular tests and DNAmt sequencing as the causative agent in the 2015–2016 malaria outbreak in RJ, wherein the patients were essentially nonresidents of foci [2]. Nevertheless, six human asymptomatic infection by *P*. *malariae* were detected by PCR in residents of Guapimirim in RJ, in 2011 [19], and a subsample of reactive local individuals for any plasmodial species revealed antibodies against erythrocytic antigens of *P*. *malariae* in 30.9%. The hypothesis of infection of NHP origin due to *P*. *brasilianum* was proposed because no index case of introduced or imported human case of *P*. *malariae* was identified in Guapimirim, and because the cases had close contact with the Atlantic forest [19]. Similar situations have been reported in neighboring states, covered by the Atlantic forest, such as SP and ES [3,4,11,51–54]. Noteworthy, *P*. *brasilianum* is a widespread and common simian malarial parasite in the Amazon [5,14] that is experimentally found to infect humans, either by inoculation of parasitized monkey blood or by the bite of infected mosquitoes [9]. High prevalence of antibodies against sporozoites antigens and erythrocytic forms of *P*. *brasilianum/malariae* in people living or frequently working in the Amazon forests (e.g., Indians, miners, settlers) of Brazil, French Guiana, and Venezuela suggested infection of this simian quartan malaria parasite in humans [55–57]. Infections by *P*. *brasilianum/malariae* in humans would be, therefore, expected to occur in RJ and other southeast states where *P*. *simium* has been described. In particular, the natural vector of both parasites is the same (*An*. *cruzii*) [5]. However, it remains unclear why malaria cases due to *P*. *brasilianum/malariae* have not been consistently reported in the state. Further, strengthening of malaria surveillance either in residents or visitors of the Atlantic forest to evaluate the zoonotic potential of *P*. *brasilianum/malariae* in South and Southeast Brazil is recommended [1].

Noteworthy, most of the *P*. *simium* and *P*. *brasilianum/malariae-*infected howler monkeys (eight of 11;73%) were from the forest coastal slope of Serra do Mar, where all autochthonous human malaria cases have been acquired [2,19]. At least two main premises may explain this geographical association: from the entomological and climatic view point, the higher relative humidity in the costal slope [58,59] may increase *An*. *cruzii* survival rates, supporting the sporogonic cycle of the *Plasmodium*. Sea moisture also favors the density of epiphyte shade bromeliads, the larval habitat of *An*. *cruzii*, and generates higher rainfall indexes [58,59], which in turn increases the amount of water accumulated in the vector larval habitats, positively influencing the mosquito density. Greater longevity and density directly influence the vector capacity of the mosquito to transmit *Plasmodium* [60,61]. Vector competence is governed by genetics of vector population, and therefore, influences *Plasmodium* transmission dynamics [61,62]. Indeed, Deane (1992) has emphasized that environmental conditions highly influence the presence and densities of Neotropical NHP hosts, bromeliads, and *An*. *cruzii*, and consequently, define the occurrence or absence of simian malaria in nearby sites. Moreover, two genetic lineages of *An*. *cruzii* with partial reproductive isolation have been recently described in Serra do Mar, one curiously occurring in the coastal region and another in the continental slopes [63]. Compared to the continental side, coastal slopes of Serra do Mar comprise a higher number of sites where people from major cities choose to reside in country houses in the forest and include ecotourism areas such as waterfalls and natural parks attracting many visitors (personal observation). As elaborated, the autochthonous human cases in the Atlantic forest in RJ have been reported mainly in the nonresidents [1,2,19]. Collectively, the environmental, entomological, ecological, and epidemiological characteristics seem to indicate that the costal slope of Serra do Mar is the riskiest place to acquire malaria of NHP origin. Protective measures such as the use of repellents and long clothes should be encouraged specifically for those who live or practice ecotourism in this slope.

During the present study, a YFV outbreak erupted in the Southeast Brazil, a region without records of this virus presence for almost 80 years [25,64]. Hundreds of epizootics of NHPs were reported, causing a significant impact on the population size of howler monkeys, an extremely susceptible host to YFV [22,65–70]. Considering the role of the howler monkeys as a reservoir of *Plasmodium* infective to humans, it is plausible to suppose that dynamics of zoonotic transmission of malaria will undergo short or mid-term changes in RJ and bordering states affected by the YFV epizooties. In this context, we postulate that the rapid decrease of *Alouatta* populations would also reduce the source of plasmodial infection to *An*. *cruzii*, which would further hamper the circulation of *Plasmodium sp*. in the Atlantic forest. Despite the short duration since the 2016–2018 YFV epizootics, records from the Brazilian Ministry of Health surveillance program seem to confirm this scenario. In fact, there was an abrupt drop in the human malaria case records between 2018 and 2019 (only one autochthonous case) [71], which was contradictory with the numbers reported between 2006 and 2014, when 4.7 cases were registered per year, on an average [2]. If the reduction of autochthonous malarial cases in the Atlantic Forest is a consequence of the *Alouatta* deaths, leading to plasmodial sources reduction, the role of howler monkeys for the occurrence of malaria in the Atlantic Forest would be reinforced.

Previous sampling efforts on examining free-living NHPs in the Southeastern Atlantic Forest over the last 30 years revealed limited geographical coverage, with samplings essentially limited to the wildlife rescues or carried out in areas close to cities, or were based on few individuals [4,38,39]. As a result, our data contribute in understanding the simian malarial parasite distribution and frequency as well as the zoonotic characteristics of autochthonous human malaria in RJ, which in turn provides assistance in shaping surveillance and control. The evidence of the NHP origin of parasites infecting humans and the widespread occurrence of anophelines vectors in the Southeast region increased the concern of the reemergence of endemic or epidemic autochthonous transmission in the region independent of the enzootic cycle [2]. However, it is not clear whether the parasitemic humans infected by the bite of *An*. *cruzii* carrying esporozoites of *P*. *simium* acquired from howler monkeys could be a source of infection to *An*. *cruzii* or any other malarial vector present in the region. It is known that *P*. *simium* infected humans usually display scanty to null parasitemia, and can be cured spontaneously in few days without treatment and any relapse; moreover, the molecular detection of parasites during treatment follow-ups has been described [2,5]. Besides, all autochthonous human cases of benign tertian malaria detected for decades in Southeast have reported recent contact with the *P*. *simium* enzootic forest, and no secondary transmission directly derived from a human infected in the zoonotic cycle has ever been detected outside the sylvatic foci. These epidemiological and parasitological profiles appear to indicate that humans are not a source of *P*. *simium* infection for mosquitoes. Thus, determining vector competence of *An*. *cruzii* and other traditional human malaria vector occurring in the Southeast region (e.g., *An*. *darlingi*, *An*. *Aquasalis*, and *An*. *albitarsis*) for transmitting *P*. *simium* and *P*. *brasilianum* between humans and from NHPs and humans and vice-versa is imperative.

## Supporting information

S1 Table Number of examined NHPs, by species, habitat, and capture method. (DOCX)
